# RNF31 represses cell progression and immune evasion via YAP/PD-L1 suppression in triple negative breast Cancer

**DOI:** 10.1186/s13046-022-02576-y

**Published:** 2022-12-29

**Authors:** Huijie Yang, Min Xue, Peng Su, Yan Zhou, Xin Li, Zhongbo Li, Yan Xia, Chenmiao Zhang, Mingxi Fu, Xiuxia Zheng, Guosheng Luo, Tian Wei, Xinxing Wang, Yinlu Ding, Jian Zhu, Ting Zhuang

**Affiliations:** 1 Xinxiang Key Laboratory of Tumor Migration and Invasion Precision Medicine, School of Laboratory Medicine, Xinxiang Medical University, Xinxiang, 453003 Henan Province People’s Republic of China; 2 Molecular Biology Laboratory, First People’s Hospital of Shangqiu, Shangqiu, City, 476000 Henan Province People’s Republic of China; 3 Department of Pathology, Shandong University Qilu Hospital, Cheeloo College of Medicine, Shandong University, Jinan City, Shandong Province People’s Republic of China; 4 Department of General Surgery, The Second Hospital, Cheeloo College of Medicine, Shandong University, Jinan, Shandong Province 250033 People’s Republic of China; 5 The Affiliated people’s Hospital of Xinxiang Medical University, Xinxiang, 453003 Henan Province People’s Republic of China; 6 Department of Breast Surgery, The First Affiliated Hospital of Zhengzhou University, Zhengzhou, Henan Province 450052 People’s Republic of China

**Keywords:** TNBC, RNF31, YAP, PD-L1, Stability, Immune evasion

## Abstract

**Background:**

Recently genome-based studies revealed that the abnormality of Hippo signaling is pervasive in TNBC and played important role in cancer progression. RING finger protein 31 (RNF31) comes to RING family E3 ubiquitin ligase. Our previously published studies have revealed RNF31 is elevated in ER positive breast cancer via activating estrogen signaling and suppressing P53 pathway.

**Methods:**

We used several TNBC cell lines and xenograft models and performed immuno-blots, QPCR, in vivo studies to investigate the function of RNF31 in TNBC progression.

**Result:**

Here, we demonstrate that RNF31 plays tumor suppressive function in triple negative breast cancer (TNBC). RNF31 depletion increased TNBC cell proliferation and migration in vitro and in vitro. RNF31 depletion in TNBC coupled with global genomic expression profiling indicated Hippo signaling could be the potential target for RNF31 to exert its function. Further data showed that RNF31 depletion could increase the level of YAP protein, and Hippo signaling target genes expression in several TNBC cell lines, while clinical data illustrated that RNF31 expression correlated with longer relapse-free survival in TNBC patients and reversely correlated with YAP protein level. The molecular biology assays implicated that RNF31 could associate with YAP protein, facilitate YAP poly-ubiquitination and degradation at YAP K76 sites. Interestingly, RNF31 could also repress PDL1 expression and sensitive TNBC immunotherapy via inhibiting Hippo/YAP/PDL1 axis.

**Conclusions:**

Our study revealed the multi-faced function of RNF31 in different subtypes of breast malignancies, while activation RNF31 could be a plausible strategy for TNBC therapeutics.

**Supplementary Information:**

The online version contains supplementary material available at 10.1186/s13046-022-02576-y.

## Highlights

1. RNF31 restrains cancer cell progression in TNBC.

2. RNF31 regulates YAP protein stability via promoting YAP K48-linked poly-ubiquitination.

3. RNF31 represses immune evasion via suppressing YAP/PD-L1 axis in TNBC.

**Supplementary Information:**

The online version contains supplementary material available at 10.1186/s13046-022-02576-y.

## Background

Breast cancer is the leading cause for female malignancies and ranks NO.2 in women cancer mortality [1]. According to the molecular classification, breast cancer can be divided into Luminal A type, Luminal B type, HER2 over-expression (Human Epidermal Growth Factor Receptor 2) and triple negative breast cancer (TNBC). TNBC accounts for about 15 to 20% of all breast cancers. It is the most malignant and aggressive type of breast cancer and is prone to early recurrence and metastasis [2, 3]. Importantly, since a lack of effective therapeutic inhibitors, such as tamoxifen for luminal types of breast cancer and Herceptin for HER2 over-expression breast cancer, TNBC treatment is still highly dependent on chemotherapy [4]. Therefore, the understanding of underlying mechanisms for TNBC carcinogenesis and progression is especially significant for the development of novel therapeutic strategies [5]. The definition of TNBC is based on an exclusive conception, which is none-hormone receptors and none-HER2 expression breast cancers [5]. Thus, TNBC includes a series of unclassified and highly heterogeneous breast malignancies, making it a big problem to discover certain molecular markers as therapeutic targets.

RNF31 (RING finger protein 31; HOIP; ZEBRA) was firstly discovered in 2004 from breast cancer MCF-7 cells, which contained several functional domains including ZNF-RBZ domain, UBA (Ubiquitin binding associated) domain and RBR (RING-IBR-RING) domain [6]. The RBR domain was regarded as the functional domain to exert its ubiquitin ligase activity. RNF31 was widely expression in several human tissues including muscle and heart. RNF31 whole knockout mice could lead to embryonic death due to the failure of angiogenesis, indicating its important role in tissue homeostasis [7]. RNF31 has been shown to be involved in the progression of several human malignancies. For example, RNF31 could form the linear ubiquitin assembly complex (LUBAC) together with RBCK1, SHARPIN and facilitate the activation of NF-κB and B cell lymphoma progression [8]. Furthermore, RNF31 also protects B cells from DNA damage-induced cell death [9]. Besides, RNF31 could facilitate pancreatic tumor evading from CD8+ T cell killing [10]. In breast cancer, our previously published research studies have revealed that RNF31 could facilitate ER positive breast cancer tumor growth via inhibiting P53 signaling and activating estrogen signaling [11, 12].

Since the oncogenic function of RNF31 has been explored in ER positive breast cancer, we further focus on the function of RNF31 in TNBC cells. Surprisingly, RNF31 depletion didn’t lead to TNBC cell inhibition, but showed even more aggressive phenotypes, which indicated it as a tumor suppressive player in TNBC. Our RNA sequencing data revealed the possible link between RNF31 and Hippo/YAP axis in TNBC. Further molecular biology studies demonstrated that YAP was a substrate for RNF31 to exert its E3 ubiquitin ligase function. Our study revealed a tumor suppressive role of RNF31 to YAP protein ubiquitination and stability, which controlled the transcriptional regulation of Hippo target genes and TNBC progression.

In recent years, with the great success of cell surface programmed death receptor-1 (PD-1) monoclonal antibody therapy in melanoma, immunotherapy has become central issue in malignant tumors therapy [13]. Breast cancer progression is related to tumor immune microenvironment and immune escape. The immune checkpoint blockade molecules, such as PD-L1, repress CD8 + T-cell cytotoxicity to facilitate immune evasion [14]. PD-L1 is a hinge immune checkpoint molecule, which together with PD-1 exerts a significant function in the clinics for TNBC [15, 16]. Previously published studies have revealed that PD-L1 is regulated by various factors. For example, c-MYC can directly bind to the promoter region of PD-L1 to increase its transcription [17], while YAP/TEAD promotes the transcription of PD-L1 by binding to the enhancer region of PD-L1 [18–20]. Our studies further certified RNF31 could repress immune evasion via YAP/PD-L1 axis suppression in Triple Negative Breast Cancer.

## Materials and methods

### Cell lines

The cell lines MDAMB231, BT549, 4 T1 and the HEK293T were purchased from the American Type Cell Culture Collection (ATCC). MDAMB231 and HEK293T cells were cultured with DMEM (41965, Life Technologies). BT549 and 4 T1 cells were cultured with RPMI-1640 (42401, Life Technologies). All cell lines were supplemented with 10% FBS (10270, Life Technologies) and maintained with 1% penicillin/streptomycin (C0222, Beyotime) with 5% CO2 at 37 °C, and were digested and passaged according to ATCC recommendations. The PowerPlex 21 system was used to profile all cell lines and validate their certification and authenticity.

### Plasmids, siRNA and reagents

Plasmids were transfected with Lipo2000 (11,668,019, Thermo) based on manufacturer’s instructions. siRNA was transfected with Lipofectamine RNAiMAX (13,778,150, Thermo) based on manufacturer’s instructions. The Flag-RNF31, HA-Ub, HA-Ub-K48, HA-Ub-K63, HA-K48R and HA-Ub-KO plasmids were obtained from plasmid were used in our previous study [11]. The RNF31 deletion constructs were purchased from HANBIO Biological (Shanghai, China). The Myc-YAP, YAP truncation mutants, lysine mutation YAP and TEAD-reporter plasmids were kindly gifted by Xiaofeng Zhou [21]. The pLVX-RNF31 were constructed by cloning an RNF31 PCR fragment into the pLVX-EF1a-ZsGreen vector digested via EcoRI and NotI by T4 DNA ligase. We cloned an 800 bp fragment of human genomic DNA around the YAP–TEAD binding site in the 13-kb upstream from the PD-L1 gene into pGL4.26 luciferase vector (Promega). The human RNF31 target sequences (siRNA, GenePharma) were as follows: siRNA#1: GCG AUU AUA UGG CUA CAC A, UGU GUA GCC AUA UAA UCG C； siRNA#2: GGC GUG GUG UCA AGU UUA A, UUA AAC UUG ACA CCA CGC C；.

siRNA#3: UGAACAUCCUGGAGAAAUAUU, UAUUUCUCCAGGAUGUUCAUU.

The human YAP target sequence (siRNA, GenePharma) was as follows: GCU CAU UCC UCU CCA GCU UTT; AAG CUG GAG AGG AAU GAG CTT. The negative control (siRNA, GenePharma) was as follows: UUC UCC GAA CGU GUC ACG U; ACG UGA CAC GUU CGG AGA A. Some key reagents are provided below: Verteporfin were purchased from MCE (Cat. No. HY-B0146); Adezmapimod (SB 203580) were obtained from MCE (Cat. No. HY-10256); MG132 and Cycloheximide (CHX) were purchased from MCE (Cat. No. HY-13259) and MCE (Cat. No. HY-12320).

### Quantitative real-time PCR

Quantitative real-time PCR were measured as described [22]. Primers used were as follows: RNF31 5′-GAG CCC CG AAA CTA CCT CAA C′; 5′-CTT GAC ACC ACG CCA GTA CC-3′. CTGF: 5′-CAG CAT GGA CGT TCG TCT G-3′; 5′-AAC CAC GGT TTG GTC CTT GG-3′. CYR61: 5′-GGT CAA AGT TACC GGG CAG T-3′; 5′- GGA GGC ATC GAA TCC CAG C-3′. 36B4: 5′-CGA CCT GGA AGT CCA ACT AC-3′; 5′-ATC TGC TGC ATC TGC TTG-3′.

### Coimmunoprecipitation (co-IP) assay and Western blot

Cells were lysed with Western and IP lysis buffer (P0013J, Beyotime) with a protease inhibitor cocktail (Roche). Total cell lysates were incubated with 30 μl of Protein Agarose (Beyotime, P2012) and IgG (Beyotime, 1:50) for preclearing 2 h at 4 °C, and immunoprecipitation was then performed with an indicated antibody for 4 h at 4 °C. As a negative control, Rabbit IgG (Beyotime, A7016, 1:50) or Moues IgG (Beyotime, A7028, 1:50) was used. Separation of proteins by SDS-polyacrylamide gel electrophoresis (PAGE) and transfer to nitrocellulose membranes (Millipore) were carried out. The antibodies were used as follows: anti-RNF31 (ab125189, Abcam, 1:2000); anti-HA (MMS-101R, COVANCE, 1:2000); anti-Flag (20543–1-AP, Proteintech, 1:2000); anti-YAP (14,074, Cell Signaling Technology, 1:2000; SC-101199, Santa Cruz, 1;500); PD-L1 (13,684, Cell Signaling Technology; 66,248–1-Ig, Proteintech); anti-Tubulin (11224–1-AP, Proteintech, 1:5000); anti-Histone-H3 (17168–1-AP, Proteintech, 1:1000) anti-Myc (Ab9106, Abcam, 1:2000); and anti-β-actin (8H10D10, Cell Signaling Technology, 1:10000). AffiniPure goat anti-mouse peroxidase-conjugated antibody IgG-HRP (A0216, Beyotime, 1:5000) or goat anti-rabbit IgG-HRP (A0208, Beyotime, 1；5000) secondary antibodies was incubated with the membranes after three washes with PBST. The signals were detected with the ECL Kit (Meilunbio, #MA0186).

### Transwell, EdU and wound healing assay

MDAMB231 cells and BT549 cells were transfected with siRNF31 or sControl. The method of transwell assay, EdU assay and wound healing assay were measured as described [23, 24].

### Immunofluorescence assay

4% paraformaldehyde was used to fix MDAMB231 cells for 15 min, 0.25% Triton X-100 to permeabilize them for 15 min, and 3% BSA to block them for 1 h at room temperature. The rabbit anti-RNF31 antibody (ab125189, Abcam, 1:400), mouse anti-YAP antibody (SC-101199, 1:200) and mouse anti-PD-L1 antibody (1:400, 66,248–1-Ig, Proteintech) were used. The Alexa Flour 647 (Invitrogen) anti-mouse or Alexa Flour 488 anti-rabbit secondary antibodies (Invitrogen). The nuclei were stained with DAPI (Beyotime). Samples with only secondary antibodies and no primary antibodies were used as negative controls. Images were captured with a Nikon A+ laser scanning confocal system.

### Immunohistochemistry

The streptavidin-peroxidase-biotin (SP) immunohistochemical method was used to measure RNF31 and YAP protein expression in 52 TNBC breast tissues. For primary antibodies, sections of tissues were incubated with RNF31 (ab125189, Abcam, 1:200) or YAP (14,074, Cell Signaling Technology, 1:200) antibodies overnight at 4 °C, while sections of xenografts from BALB/c mice with rat monoclonal anti-CD8 (eBioscience, 1:200). Immunoreactivity was measured as depicted in previous paper [25]. This usage of clinical samples was reviewed and approved by the Ethical Board at Shandong University with written informed consent from all the patients.

### Flow cytometric analyses

MDA-MB-231 and BT549 cells were transfected with siRNF31 or siControl. 48 hours after transfection. For apoptosis analysis, the cells were treated following the Apoptosis Detection Kit (A211–01, Vazyme). For CD44/CD24 analysis, the cells were digested and then washed with PBS (with 1% FBS) for 3 times, then resuspension in 100 μl PBS, and then stained with anti-CD44-PE (BD, 1:500) and anti-CD24-FITC (BD, 1:500). The samples were then washed by PBS 3 times and finally re-suspended in 400 μl PBS. For membrane PD-L1 analysis, the cells were digested and stained with PD-L1 antibody according to PD-L1 antibody (13,684, Cell Signaling Technology). The CD45 analysis was performed on tumor tissue samples from mice after they were washed with PBS, minced, and treated with a digestive solution containing 95% RPMI 1640, 2% FBS, 1% collagenase IV, and 1% DNase I and 1% Dispase II at 37 °C. Then, single cells were centrifuged (200 rpm for 30 min) and stained with anti-mouse CD45 eFluor 450 (B220, 48–0452-82). The data was analyzed using a Beckman FACS flow cytometer.

### Tumorigenesis essay

Specific pathogen-free (SPF) conditions were maintained, with an approximately 12 h light/12 h dark cycle, and free access to food and water were provided for all animals. 6-week-old female BALB/c nude mice and BALB/c mice were purchased from Beijing Vital River Laboratory Animal Technology Co., Ltd. BALB/c nude mice (*n* = 6 for each group) were injected with 3 × 10^6^ MDAMB231 cells in 100 μl PBS subcutaneously. 5 × 10^6^ 4 T1 cells in 100 μl PBS with 50 μl Matrixgel Basement Membrane Matrix (BD Biosciences) were injected into the BALB/c mammary fat fad. Tumor growth of 4 T1 cells in BALB/c mice treated with mPD-L1 antibody ((Bio X Cell, 10F.9G2)) or IgG ((Bio X Cell) as indicated. Tumors were measured at the indicated time points (n = 6 mice per group). Tumor volume was calculated by formula volume = length× (width^2^)/2. All animal protocols in this study were approved from the Animal Care and Use Committee of Xinxiang Medical University.

### Poly-ubiquitination assay

To directly detect K48 ubiquitinated and total ubiquitinated YAP enriched in cell extracts, HA-K48 ubiquitinated or HA-Ub plasmids, Myc-YAP and Flag-RNF31 or Flag-Vectors were transfected into HEK293T cells. After 24 hours, 10 μM MG132 was added to the cells for treating 8 hours. Then, cells were lysed with Western and IP lysis buffer (P0013J, Beyotime) with a protease inhibitor cocktail (Roche). Total cell lysates were incubated with 30 μl of Protein Agarose (Beyotime, P2012) for preclearing 2 h at 4 °C, and immunoprecipitation was then performed with anti-YAP antibody (14,074, Cell Signaling Technology) or Rabbit IgG (Beyotime, A7016) for 4 h at 4 °C. Western blot was performed with anti-HA antibody to detect YAP total polyubiquitinated or YAP K48 polyubiquitinated.

### Chromatin immunoprecipitation (ChIP)

By reviewing Zanconato; et al. preserved ChIP-Seq data (GSE66081 [26]) and referring to Min Hwan Kim; et al. [18]. We designed a pair of primers for ChIP analysis targeting the narrow peak of TEAD4 binding 13-kb upstream of PD-L1 transcription start site. Primers were used as follows: 5′-CAT CGG GAT TAC CAC GCT GA-3′ and 5′-TTC GTT CCA TTA GAG CGC GT-3′. ChIP assay was executed in indicated MDAMB231 cells according to the Magna ChIP A/G kit (17–10,085, Sigma-Aldrich) instructions. Sheared chromatin was immunoprecipitated by IgG or anti-Myc antibody at 4 °C for 12 h. YAP binding with the 13-kb upstream site was detected by quantitative PCR using SYBR Green qPCR assay (639,676, TaKaRa).

### Dual-luciferase reporter assay

The Dual-Luciferase Reporter Assay System (E1910, Promega, United States) was used to detect Luciferase activity. The cells were transfected with the TEAD luciferase reporter or pGL4.26-YAP–TEAD binding site and the Renilla plasmid. After 24 hours, cells were lysis, and luciferase activity was detected. A GloMax-Multi Jr. (Promega-GloMax Promega, USA) was used to detect luciferase activity.

### Publicly available clinical data analysis

Analysis of the expression of RNF31 was carried out with data for 1080 breast cancer samples from the TCGA database. Expression in ER positive, HER2-positive, and triple-negative breast cancer tissues and normal tissues were analyzed with GraphPad Prism 9. Analysis of RNF31 and YAP with clinical prognosis was analyzed by KMPLOT database (https://kmplot.com). RNF31 RNA-seq data were downloaded from the GEO datasets (GSE218406). For CORDENONSI YAP CONSERVED SIGNATURE gene sets were used and downloaded from the GSEA Molecular Signatures Database. GSEA was implemented by GSEA 4.3.2 software.

### Statistical analysis

Statistical analyses were carried out and created using GraphPad Prism 9 software. All the data are showed with the mean ± standard deviation (SD) for at least 3 independent experiments. *P* value< 0.05 was considered to indicate statistically significant using two-tailed Student’s t test and Chi-square test. *P < 0.05, **P < 0.01, ***P < 0.001.

## Result

### RNF31 restrains cancer cell progression in TNBC

**Fig. 1:**
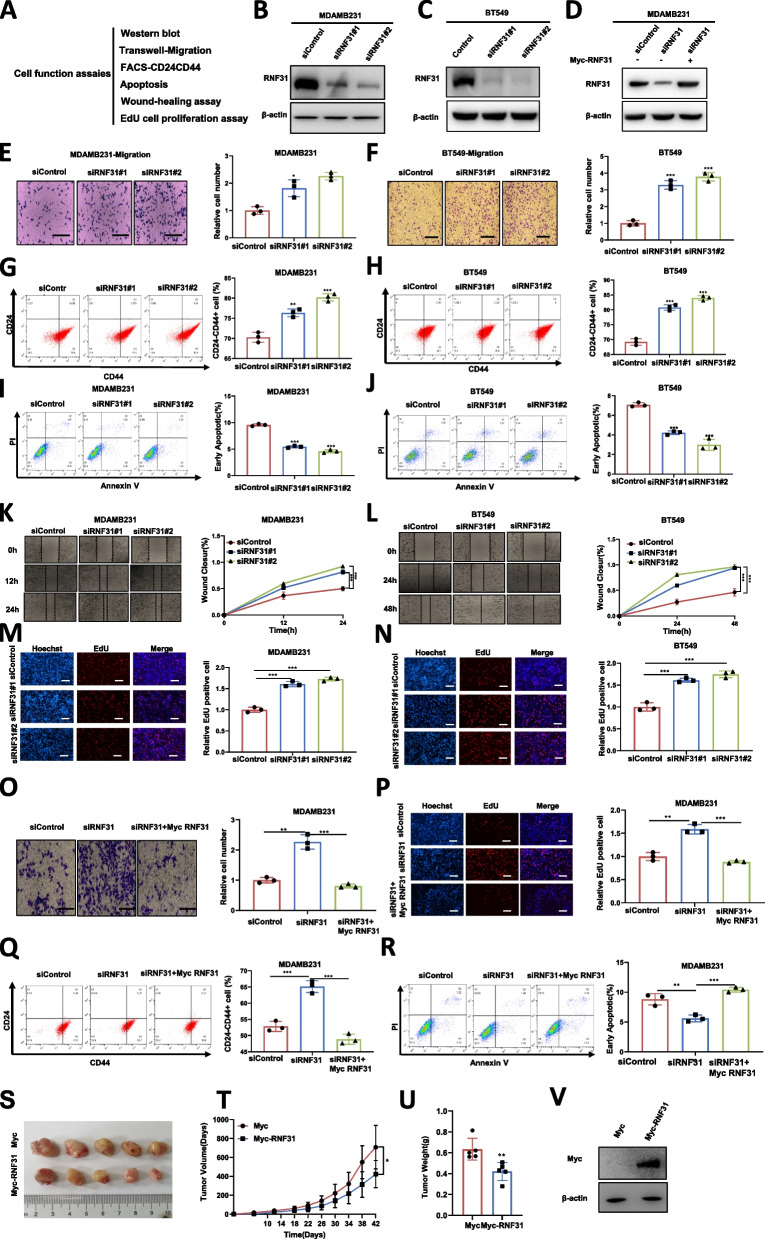
RNF31 restrains cancer cell progression in TNBC (**A**) Experimental scheme to test cell biological function. (**B-D**) Western blot detecting of RNF31 expression in MDAMB231 and BT549 cells exposed to indicated methods. (**E-F** and **O**) Transwell assay (left panel) of MDAMB231(E) and BT549(F) cells. Right panel shows quantification of transwell assay results. Scale bar 100 μm. (G-H and Q) FACS analysis (left panel) was performed on the MDAMB231 and BT549 cells to detect the proportion of CD44 + CD24-cells. Right panel shows quantification of CD44 + CD24- proportion. (I-J and R) FACS analysis (left panel) was performed on the MDAMB231and BT549 cells to detect the proportion of apoptotic cells. The cells were incubated with PI and Annexin V. Right panel shows quantification of apoptosis proportion. (**K-L**) Wound healing assay (left panel) of MDAMB231(**K**) and BT549(**L**) cells migration capability following transfected with indicated treatment. Quantification of wound closure at the specified points in time. Right panel shows quantification of wound healing results. (**M-N** and **P**) Representative images (left panel) of EdU assays in MDAMB231and BT549 cells transfected with indicated treatment. EdU-positive cells, red; cell nuclei, blue. Right panel shows quantification of Edu results. Scale bar 100 μm. (**S**) Representative image of tumor derived from BALB/c nude mice injected with Myc or Myc-RNF31 stably transfected MDAMB231 cells is followed as indicated. (**T-U**) The tumor volume (**T**) and weight (**U**) in BALB/c nude mice subcutaneously inoculated Myc or Myc-RNF31 stably transfected MDAMB231 cells. (**V**) Western blot detecting of RNF31 expression in tumor tissues in nude mice injected with Myc or Myc-RNF31 stably transfected MDAMB231 cells. The results are representative of 3 independent experiments in panel B-R. The results are representative of 5 independent experiments in panel S-U. β-actin was engineered to the internal reference for Western blot. The data are presented as mean ± SDs. *P < 0.05, **P < 0.01, ***P < 0.001 (Student’s t test)

We firstly examined several cancer cell phenotypes in two TBNC cell lines, including MDAMB231, BT549 and HS578T (Fig. 1A). The knocking down efficiency of RNF31 in MDAMB231, BT549 and HS578T cells were shown in Fig. 1B, 1C and Fig. S1A. RNF31 overexpression efficiency in MDAMB231 cells was shown in Fig. 1D. The trans-well assay showed that RNF31 depletion could enhance TNBC migration capacity in MDAMB231, BT549 and HS578T cells (Fig. 1E-1F, S1B-C). Researchers have suggested that CD44+/CD24- breast CSCs could play a dominant role in TNBC recurrence, thanks to their potent self-renewal and differentiation abilities [27]. RNF31 depletion coupled with flowcytometry analysis indicated that RNF31 depletion could increase the population of CD24−/CD44+ cells in MDAMB231, BT549 and HS578T cells (Fig. 1G-1H, S1D-E). We further investigated the effect of RNF31 in cell early apoptosis. The Propidium Iodide (PI) coupled with Annexin V staining showed that RNF31 depletion inhibited cancer cell apoptosis in MDAMB231, BT549 and HS578T cells (Fig. 1I-1J, S1F-G). RNF31 depletion further enhanced the ability of TNBC to migrate according to the wound-healing assay (Fig. 1K-1L, S1H-I). RNF31 depletion increased proliferation in MDAMB231, BT549 and HS578T cells as determined by the EdU incorporation assay (Fig. 1M-1N, S1J-K). Then we further checked RNF31 function in conjunction with siRNF31 in MDAMB231 cells. The data showed that RNF31 overexpression could bring back the protein level of YAP in RNF31 knocking down background (Fig. 1D). The trans-well assay showed that RNF31 overexpression could reduce the migration capacity in RNF31 knocking down background (Fig. 1O). The EdU staining showed that RNF31 overexpression could reduce the number of EdU positive cells in RNF31 knocking down background (Fig. 1P). Besides, in RNF31 knocking down background, RNF31 overexpression could decrease the proportion of CD44+/CD24- breast cancer cells (Fig. 1Q). In the apoptotic assay, RNF31 overexpression could increase the proportion of apoptotic cells in RNF31 knocking down background (Fig. 1R). The xenograft mouse model to study the role of RNF31 in vivo confirmed that overexpression of RNF31 could reduce the growth rate of tumors in vivo (Fig. 1S-V). Overall, all these data demonstrated the inhibitory role of RNF31 in TNBC progression.

### RNA sequence and clinical data analysis reveals the link between RNF31 and hippo pathway in TNBC

**Fig. 2:**
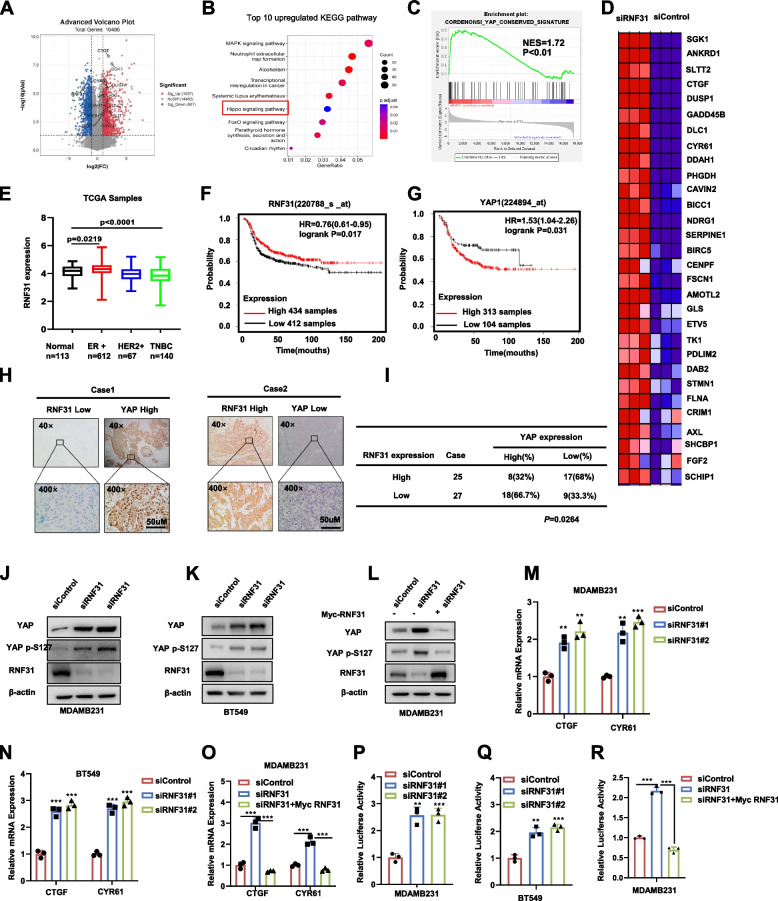
RNA sequence and clinical data analysis reveals the link between RNF31 and Hippo pathway in TNBC. **A** Volcano map of RNA-seq data from MDAMB231 cell lines treated with siControl or siRNF31. The volcanic map analysis showed that CTGF、CYR61 SGK1.et genes downstream of YAP were significantly up regulated in the RNF31 depletion group. Threshold P < 0.05 and fold change> 2 is set as screening criteria. **B** Top 10 KEGG pathway enriched by differentially up-regulated genes in RNA-seq data of RNF31 depletion group. Threshold *P* < 0.05. **C** Gene set enrichment analysis (GSEA) of RNA-seq data from MDAMB231 cell lines treated with siControl or siRNF31. The gene sets of CORDENONSI YAP CONSERVED SIGNATURE were enriched in the RNF31 depletion group. Threshold P < 0.05. **D** Heatmap of YAP related genes in RNA-seq data from MDAMB231 cell lines treated with siControl or siRNF31. Threshold P < 0.05 and fold change> 1.5. **E** The expression distribution of RNF31 in Luminal A, Luminal B, HER2 positive and TNBC tissues and normal tissues from TCGA database (https://tcga-data.nci.nih.gov). **F-G** Kaplan-Meier map of progression free survival of RNF31 and YAP in triple negative breast cancer patient. (http://kmplot.com/analysis/). **H-I** Immunohistochemistry (IHC) detecting RNF31 and YAP expression in TNBC tissues. Scale bar 50 μm. Chi-square test to detect of RNF31 correlation with YAP in 52 TNBC tumor samples (**I**). **J-L** Western blot results displaying the protein level of YAP, YAP P-S127 and RNF31 in cell lines transfected with either indicated treatment. **M-O** RT–qPCR results of CTGF and CYR61 mRNA expression in cell lines transfected with either indicated treatment. **P-R** Measurement of TEAD transcriptional activity in cells using luciferase assays using reporters that contain tandem TEAD binding sites. The results are representative of 3 independent experiments in panel J-R. β-actin was engineered to the internal reference for Western blot. The data are presented as mean ± SDs. **P < 0.01, ***P < 0.001 (Student’s t test)

To analysis the potential regulation mechanism of RNF31 in TNBC, we depleted RNF31 in MDAMB231 cells (GSE218406). The volcano plot analysis indicated RNF31 depletion significantly facilitated Hippo pathway classical target genes expression, including CTGF, SGK1 and CYR61 .et (Fig. 2A). Our RNA sequence data showed that in MDAMB231 cells RNF31 could increase CTGF and CYR61 mRNA level (3.87 folds and 3.1 folds respectively, fig. S2A). The top enriched pathways include MAPK pathway, Neutrophil extracellular trap formation pathway, Alcoholism pathway, Systemic lupus erythematosus pathway, Transcriptional mis-regulation in cancer and Hippo pathway (Fig. 2B). However, Neutrophil extracellular trap formation pathway, Alcoholism pathway and Systemic lupus erythematosus pathway, do not relevant to breast cancers, which sometimes happens that certain pathways do not fit to the research models. For the other two items including MAPK pathway and Transcriptional mis-regulation in cancer, we think these two pathways are so general for cancers and a lack of specificity. As we know, almost every regulation mechanism in cancers could cause the change of MAPK pathway and transcriptional regulation. Thus, Hippo signaling is the specific pathway left, which we can dig into. Besides, we did validate the function of RNF31 in MAPK pathway, which showed that phosphor-P38 was increased by RNF31 depletion (Supplementary Fig. 2B). The specific inhibitor of p38 MAPK SB203580 could at least partially rescue the progression phenotypes, which were caused by RNF31 depletion (Supplementary fig. 3H-M).

The GESA (Gene Enrichment Signature Analysis) showed that the activated YAP conserved gene signature was globally increased in RNF31 depletion condition, NES = 1.72 (Fig. 2C). The heat-map from RNF31 depletion showed numerous Hippo target genes were promoted under siRNF31 conditions in MDAMB231 cells (Fig. 2D). Interestingly, RNF31 was elevated in ER+ breast cancer, but its expression was decreased in TNBC compared with normal breast tissue in TCGA database (Fig. 2E), which consistent with our previous study [11, 12]. We further analyzed the expression of RNF31 with relation to survival in TNBC patients, which showed that RNF31 expression correlated with longer relapse-free survival (RFS) in TNBC, while YAP correlated with poor RFS in TNBC (Fig. 2F-2G). Further immunohistochemistry analysis based on 52 cases of TNBC samples showed the reverse protein correlation between RNF31 and YAP (Fig. 2H-2I).

We further depleted RNF31 in MDAMB231, BT549 and HS578T cells to confirm its biological link to Hippo signaling. The data showed that RNF31 depletion could increase both YAP total protein level and phosphorylated YAP protein level (S127) (Fig. 2J, K, S2C). The gene expression analysis showed that RNF31 depletion could further increase Hippo target gene expression, including CTGF and CYR61 in MDAMB231, BT549 and HS578T cells (Fig. 2M, N, S2D), while RNF31 overexpression could inhibit these gene expression (Fig. 2O). The Luciferase reporter experiment showed that RNF31 depletion in MDAMB231, BT549 and HS578T cells increased TEAD response element activity (Fig. 2P, Q, S2E), while RNF31 overexpression inhibited TEAD response element activity in MDAMB231 cells (Fig. 2R). Besides, we examined the YAP mRNA level under RNF31 depleted conditions in three TNBC cell lines. The data showed that RNF31 had no effect on YAP mRNA level, which might indicate that RNF31 affected YAP protein level via post-translational regulation (Supplementary Fig. 2F-H).

### RNF31 restrains TNBC cell progression via hippo/YAP axis

**Fig. 3:**
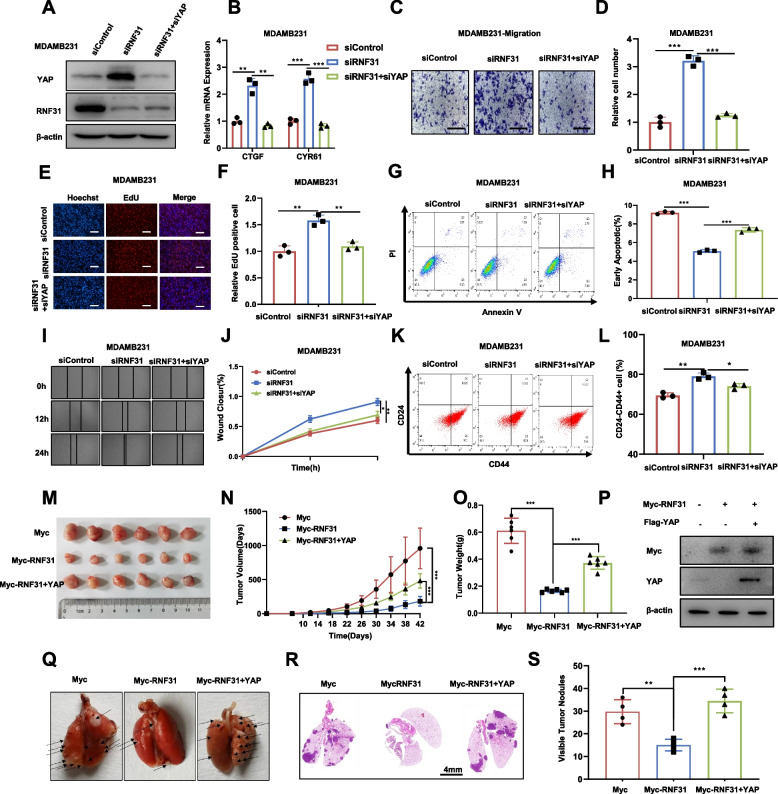
RNF31 restrains TNBC cell progression via Hippo/YAP axis. **A** Western blot detecting of YAP and RNF31 expression in MDAMB231 cell exposed to siControl or siRNF31 or siRNF31 + siYAP for 48 h, respectively. **B** RT–qPCR results of CTGF and CYR61 mRNA expression in MDAMB231 cell line exposed to indicated treatment. **C-D** Transwell assay (left panel) of MDAMB231 cells transfected with indicated treatment. Right panel shows quantification of transwell assay results. Scale bar 100 μm. **E-F** Representative images (left panel) of EdU assays in MDAMB231 cell transfected with indicated treatment. EdU-positive cells, red; cell nuclei, blue. Right panel shows quantification of Edu results. Scale bar 100 μm. **G-H** FACS analysis (left panel) was performed on the MDAMB231 cell to detect the proportion of apoptotic cells. **I-J** Wound healing assay (left panel) of MDAMB231 cell migration capability. **K-L** FACS analysis (left panel) was performed on the MDAMB231 cell to determine the proportion of CD44 + CD24-cells. **M** Representative image of tumor derived from BALB/c nude mice injected with indicated stably transfected MDAMB231 cells. **N-O** The tumor volume (**N**) and weight (**O**) in BALB/c nude mice subcutaneously inoculated with indicated stably cells. **P** Western blot detecting of YAP and RNF31 expression in tumor tissues in BALB/c nude mice injected with indicated stably cells. **Q** Representative image of In vivo lung metastasis of indicated MDAMB231 cells. **R-S** Representative HE stains pictures of lung sections of indicated MDAMB231 cells. Arrows indicate metastatic nodules (**R**). Quantification of metastatic lung nodules (**S**). The results are representative of 3 independent experiments in panel A-L. The results are representative of 6 independent experiments in panel M-Q. The results are representative of 4 independent experiments in panel Q-S. β-actin was engineered to the internal reference for Western blot. The data are presented as mean ± SDs. *P < 0.05, **P < 0.01, ***P < 0.001 (Student’s t test)

To confirm the biological link between pathway regulation and cancer phenotypes, we further carried out several rescue experiments. The immuno-blotting data showed that RNF31 knocking down enhanced the protein level of YAP, which could be brought back by further YAP depletion (Fig. 3A). The QPCR data indicated that the Hippo target genes, which were increased by RNF31 depletion, could be rescued by further YAP depletion (Fig. 3B). In the trans-well assay, RNF31 depletion enhanced invasion capacity in MDAMB231 cells, which effect could be partially rescued by further YAP depletion (Fig. 3C-D). In the EdU assay, RNF31 depletion increased the numbers of EdU positive cells, which could be further reduced via YAP depletion (Fig. 3E-3F). RNF31 depletion decreased cell early apoptosis in MDAMB231 cell, while further YAP silencing could further increase cell apoptosis (3G-3H). The wound healing assay showed that the migration capacity was significantly promoted in MDAMB231 cells after RNF31 depletion, which effect could be partially rescued by further YAP knocking down (Fig. 3I-3J). RNF31 silencing could also promote the proportion of CD24−/CD44 + cells, which could be rescued by YAP depletion (Fig. 3K-3L).

We further utilized different concentration of verteporfin (VP) to carry out a series of assays. The data showed that verteporfin could effectively decrease YAP protein level in dose dependent manner (Supplementary fig. 3A). In the trans-well assay, verteporfin effect could at least partially reverse the increased migrated cells in dose dependent manner, which was caused by RNF31 silence (Supplementary fig. 3B-C). In the apoptosis assay, we can observe that RNF31 could decrease the number of apoptotic cells, while verteporfin treatment could at least partially reverse this effect (Supplementary fig. 3D-E). In the EdU incorporation assay, we found that verteporfin effect could at least partially reverse the increased number of proliferation cells caused by RNF31 silence (Supplementary fig. 3F-G).

The xenograft mice model indicated that RNF31 overexpression could inhibit TNBC tumor growing in MDAMB231 cells, while further YAP overexpression could at least partially rescue the growth inhibition caused by RNF31 (Fig. 3M-3O). The tumor lysis confirmed the RNF31 was overexpressed, while YAP expression was inhibited in TNBC tumors (Fig. 3P). Besides, in the in vivo metastasis assay, RNF31 overexpression could significantly reduce the lung metastasis, which function could be partially rescued by YAP over-expression in MDAMB231 cells (Fig. 3Q-3S).

### RNF31 interacts with YAP and inhibits its protein stability

**Fig. 4:**
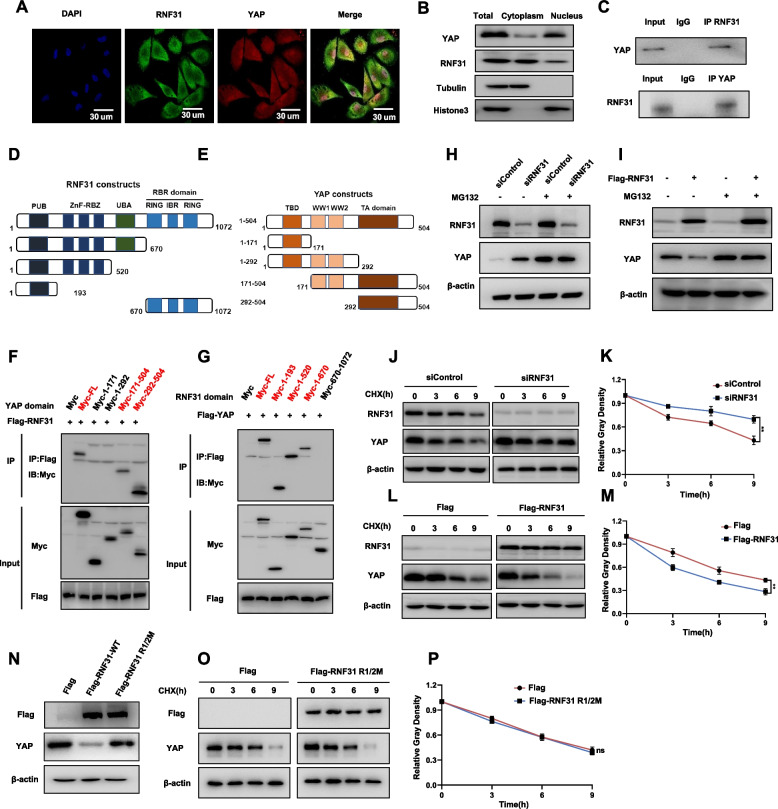
RNF31 interacts with YAP and inhibits its protein stability. **A** Immunofluorescence imaging of RNF31 (Green), YAP (Red) and DAPI (blue) in MDAMB231 cell, scale bar 30 μm. **B** Western blot detecting of YAP and RNF31 protein localized in the MDAMB231 cell. Subcellular protein fractionation kit was used for cytoplasm and nucleus separation. Tubulin and Histone3 were engineered to cytoplasm and nucleus controls. **C** Co-IP assay revealed the association between endogenous RNF31 and YAP protein in MDAMB231 cell. **D-E** Schematic diagram to show wild-type and truncated RNF31 and YAP constructs used in this study. **F** Representative immunoblots to show the interaction between RNF31, and WT or truncated YAP as indicated assessed by immunoprecipitation (IP) with RNF31 (anti-Flag). **G** Representative immunoblots to show the interaction between YAP and WT or truncated RNF31 as indicated assessed by immunoprecipitation (IP) with YAP (anti-Flag). **H-I** Western blot detecting of YAP and RNF31 protein level in MDAMB231 and HEK293T cells in indicated treated. 10 μM MG132 was added to cells for 8 h before fractured for western-blot assays. **J-M** and **O-P** Western blot detecting of YAP protein half-life in MDAMB231 and HEK293T cell. 100 μmol/L CHX was added to cells for indicated time periods before fractured for western-blot assays. **N** Western blot detecting of YAP and RNF31 protein level in HEK293T cell transfected with either Flag or Flag-RNF31 or Flag-RNF31 R1/2M plasmid. The results are representative of 3 independent experiments. β-actin was engineered to the internal reference for Western blot. The data are presented as mean ± SDs. **P < 0.01 (Student’s t test)

We further explored the localization of RNF31 and YAP in TNBC cells by Immunofluorescence assay, which showed that RNF31 was mainly localized in the cytosol, while YAP protein located both in the cytosol and nucleus (Fig. 4A). This conclusion was further confirmed by the nucleus-cytosol separation assay (Fig. 4B). In order to evaluate if RNF31 could affect YAP translocation or nuclear/cytosol localization, we utilized cytosol/nuclear separation assay in both vehicle or siRNF31 treated cells. The data showed that RNF31 depletion could increase YAP protein level both in the cytosol and nuclear. However, the ratio of nuclear/cytosol was not changed, which might indicate that RNF31 has little or no effect on YAP localization in MDAMB231 cells (Supplementary Fig. 4A-B). The co-immunoprecipitation assay illustrated the association between RNF31 and YAP in TNBC cells (Fig. 4C). Furthermore, our data also showed that RNF31 could associate with YAP but not TAZ (Supplementary Fig. 4C).

The RNF31 protein is composed of PUB domain, ZnF-RBZ domain, UBA domain and RBR (RING-in-between-RING) domain, while YAP protein is comprised of TA domain (Transcription activation domain), WW domain (WW1 domain and WW2 domain) and TBD domain (TEAD binding domain) (Fig. 4D-4E). We made these deletion constructs and analyzed the interaction domains for each protein. The co-immunoprecipitation assay illustrated that TA domain was required for YAP to interact with RNF31, while RNF31 associated with YAP through its PUB domain (Fig. 4F-4G). Since RNF31 was an E3 ubiquitin ligase, we further explored its effect on YAP protein stability. We utilized the proteasome inhibitor MG132 for further experiments, which illustrated RNF31 depletion could promoted YAP protein level, while such influence could be diminished by further MG132 treatment (Fig. 4H). Consistently, RNF31 overexpression decreased YAP protein level, which influence could be diminished by MG132 treatment (Fig. 4I). This might display RNF31 modulated YAP protein stability. The cycloheximide treatment showed that RNF31 depletion could greatly enhance YAP protein stability (Fig. 4J-4K). The protein half-life assay displayed that RNF31 over-expression could shorten the stability of YAP protein (Fig. 4L-4M). Since the cysteine residues at 699 and 702 sites were required for the ubiquitin ligase activity of RNF31, we utilized the ligase deficient RNF31 R1/2M [11]. The immuno-blotting data showed that R1/2M form of RNF31 could neither inhibit YAP protein level, nor shorten YAP protein stability as RNF31 wild type did (Fig. 4N-4P).

### RNF31 regulates YAP protein stability via promoting YAP K48-linked poly-ubiquitination

**Fig. 5:**
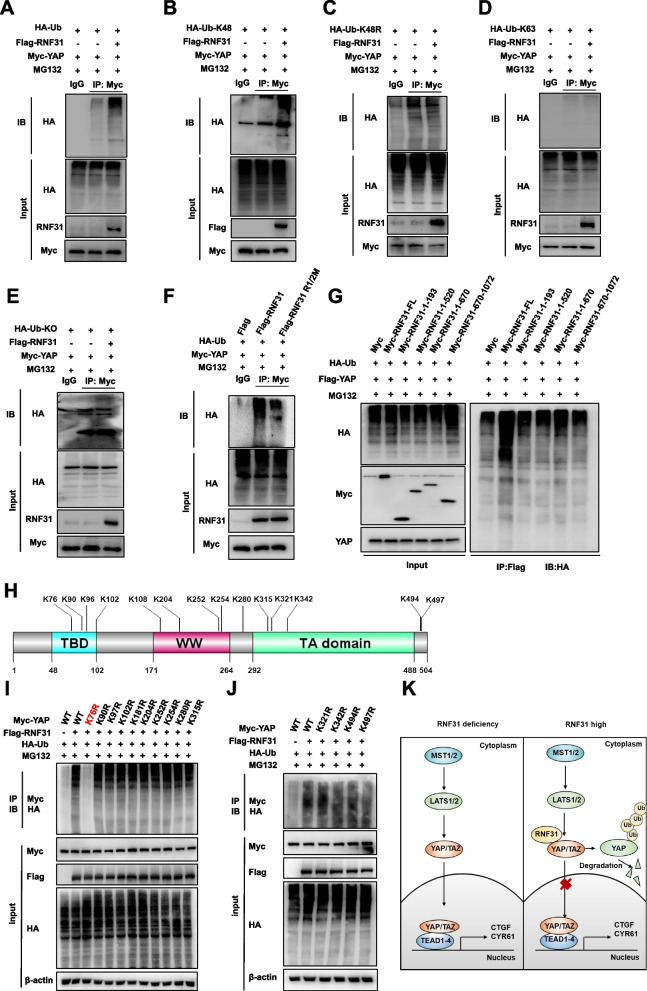
RNF31 regulates YAP protein stability via promoting YAP K48-linked poly-ubiquitination. **A** Western blot detecting of polyubiquitinated YAP was performed after coimmunoprecipitation in HEK293T cells treated in indicated. **B-E** Western blot detecting of K48-specific/K48R-specific/K63-specific/ mono-ubiquitinated YAP polyubiquitinated YAP was performed after coimmunoprecipitation in HEK293T cells treated in indicated. **F-G** Western blot detecting of polyubiquitinated YAP was performed after coimmunoprecipitation in HEK293T cells treated in indicated. **H** Schematic diagram to show lysine mutation site of YAP used in this study. **I-J** Ubiquitination of YAP at indicated multiple sites were measured by ubiquitination assay. K76 mutations (K76R) largely eliminated the ubiquitination effect of RNF31 on YAP protein. **K** Schematic illustration of RNF31 regulating Hippo/YAP signaling in TNBC progression. RNF31 protein associated with YAP and increased YAP protein degradation via inducing YAP K48-linked polyubiquitination, which repressed the Hippo/YAP axis activation and progression of TNBC cells. All the results are representative of three independent experiments

We further examined RNF31 effect on YAP ubiquitination. The immuno-precipitation experiment indicated that RNF31 over-expression could increase the overall poly-ubiquitination of YAP (Fig. 5A). Further ubiquitination assay indicated that RNF31 could enhance K48-linked poly-ubiquitination of YAP (Fig. 5B). Consistently, we further utilized the K48 mutant ubiquitin plasmid, in which RNF31 could not observe a significant regulation on the poly-ubiquitination of YAP in the ubiquitin K48 mutant background (Fig. 5C). This data clarified the poly-ubiquitination of YAP is K48 form dependent. Furthermore, we cannot observe a significant regulation on K63-linked poly-ubiqutination of YAP by RNF31 (Fig. 5D). Besides, ubiquitination assay via Ub-KO plasmid showed no significant regulation on the mono-ubiquitination of YAP by RNF31 (Fig. 5E). Further ubiquitination assay showed that the ligase deficient form of RNF31 could not efficiently promote YAP poly-ubiquitination as RNF31 wide type form did (Fig. 5F). We further overexpressed RNF31 full length or variants to see the effect on YAP protein poly-ubiquitination level in HEK293T cells, which indicated that only the full length of RNF31 was required for RNF31 to induce YAP total poly-ubiquitination (Fig. 5G). We further examined the detailed ubiquitination sites on YAP protein. Since YAP protein contains 14 lysine sites, which could be ligated, we made these lysine mutant forms. Ubiquitination based immunoprecipitation confirmed that RNF31 could promote the poly-ubiquitination of YAP at K76 sites (Fig.5H-5J). Thus, we can conclude RNF31 could associate with YAP and promote YAP poly-ubiquitination, which inhibits Hippo target gene expression and TNBC progression subsequently (Fig. 5K).

### RNF31 represses PD-L1 expression and immune evasion by suppressing hippo/YAP axis

**Fig. 6:**
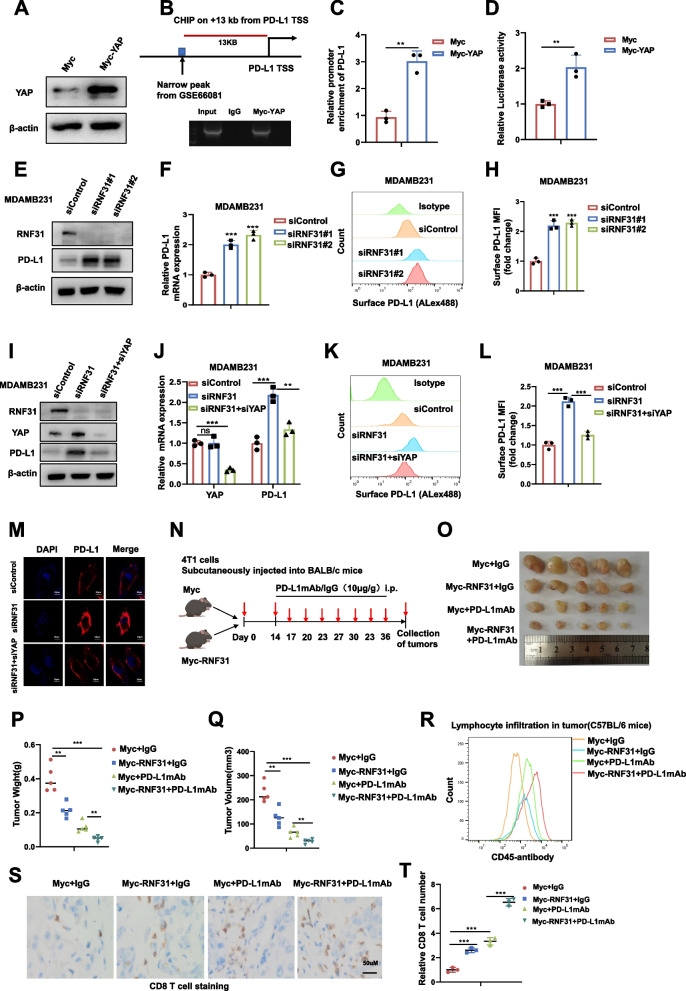
**RNF31 represses PD-L1 expression and immune evasion by suppressing Hippo/YAP axis. A** Western blot detecting of YAP protein level in MDAMB231 cell. **B** ChIP assay displaying YAP interaction with a potential enhancer located 13-kb upstream from the transcription start site of PD-L1 gene. Lysates were prepared from MDAMB231 cell transfected with Myc-YAP. YAP–TEAD interacting sequence located 13-kb upstream of PD-L1 gene was amplified by PCR after immunoprecipitation using IgG or anti-Myc antibody. **C** ChIP-qPCR data displaying relative levels of the 13-kb upstream enhancer sequence in the ChIP assay. **D** Relative luciferase activity of Myc or Myc-YAP in MDAMB231 cells transfected with luciferase vector containing the 13-kb upstream enhancer. **E** and **I** Western blot detecting of PD-L1 and RNF31 expression in MDAMB231 cell with indicated treatment. **F** and **J** RT–qPCR results of PD-L1 mRNA level with indicated treatment. **G-H** and **K-L** The cell-membrane localized PD-L1 in MDAMB231 cell with indicated treatment were analyzed by Flow cytometry using PD-L1 antibody. **M** Immunofluorescence imaging of PD-L1 (Red) and DAPI (blue) in MDAMB231 cell with indicated treatment, scale bar 20 μm. **N-T** Flowchart of In vivo experiments (**N**). Endpoint tumor images (**O**). Endpoint tumor weight (**P**). Endpoint tumor volume (**Q**). Cells digested from indicate tumor tissues in BALB/c mice were stained with anti-CD45 antibody and subjected to flow cytometric analyses (**R**). Immunohistochemical staining was performed on tumor sections with anti-CD8 antibody. Representative images are shown. Scale bars, 50 μm (**S-T**). The results are representative of 3 independent experiments in panel A-M. The results are representative of 6 independent experiments in panel N-S. β-actin was engineered to the internal reference for Western blot. The data are presented as mean ± SDs. **P < 0.01, ***P < 0.001 (Student’s t test)

PD-L1 is a hinge immune checkpoint molecule, which together with PD-1 plays an important role in the clinics for TNBC [15, 16]. Our RNA sequencing data showed that RNF31 depletion dramatically increased not only Hippo target gene expression, but also PD-L1 expression (GSE218406, FC = 2.514). Interestingly, previously published studies have revealed that PD-L1 is regulated by various factors. For example, c-MYC can directly link with the promoter region of PD-L1 to promote its transcription [17], while YAP/TEAD promotes the transcription of PD-L1 by binding to the enhancer region of PD-L1 [18–20]. We further examined the biological link if RNF31 modulated PD-L1 expression through Hippo signaling. YAP over-expression efficiency was tested in TNBC cells (Fig. 6A). The public available ChIP sequencing data (GSE66081) showed the potential YAP binding site at the upstream of 13 Kb of PD-L1 transcriptional starting site, which was confirmed by our ChIP assay in TNBC cells (Fig. 6B-C). To validate this YAP-driven transcriptional activation, we cloned a fragment 800 bp upstream of this candidate enhancer region into a luciferase vector. The luciferase activity driven by the enhancer sequence was largely promoted in YAP over-expressing MDAMB231 cells (Fig. 6D). Interestingly, RNF31 depletion in MDAMB231, BT549 and HS578T cells showed increased PD-L1 protein level and mRNA level (Fig. 6E-F, S5A-B, E-F). The FACS analysis showed that RNF31 depletion could increase the membrane PD-L1 level in MDAMB231 and BT549 cells (Fig. 6G-H, S5C-D, G-H). Besides, we further did a series of rescue assays to examine if such regulation went through Hippo pathway. The immuno-blotting displayed that RNF31 depletion could enhance PD-L1 protein level, which effect could be rescued by further YAP depletion in TNBC cells (Fig. 6I). Consistently, the QPCR data indicated that RNF31 depletion elevated PD-L1 mRNA, which was brought back by further YAP depletion (Fig. 6J). The FACS analysis also confirmed that PD-L1 membrane protein could be rescued by YAP depletion in TNBC cells (Fig. 6K-6L). The immuno-staining showed RNF31 depletion increased PD-L1 signals, which was brought back by further YAP depletion in TNBC cells (Fig. 6M). We further evaluated RNF31 effect in immuno-therapy in vivo. We utilized BALB/c mice and 4 T1 murine breast cancer cell line to carry out these experiments (Fig. 6N). The xenograft assay showed that RNF31 over-expression could inhibit TNBC tumor growth, while PD-L1 mAb treatment could further enhance tumor inhibition effect in RNF31 over-expression group (Fig. 6O-6Q). In the meanwhile, RNF31 depletion facilitated total CD45 immune cells (Fig. 6R) as well as the infiltration of CD8+ T cells (Fig. 6S-T) within the tumor mass.

## Discussion

**Fig. 7:**
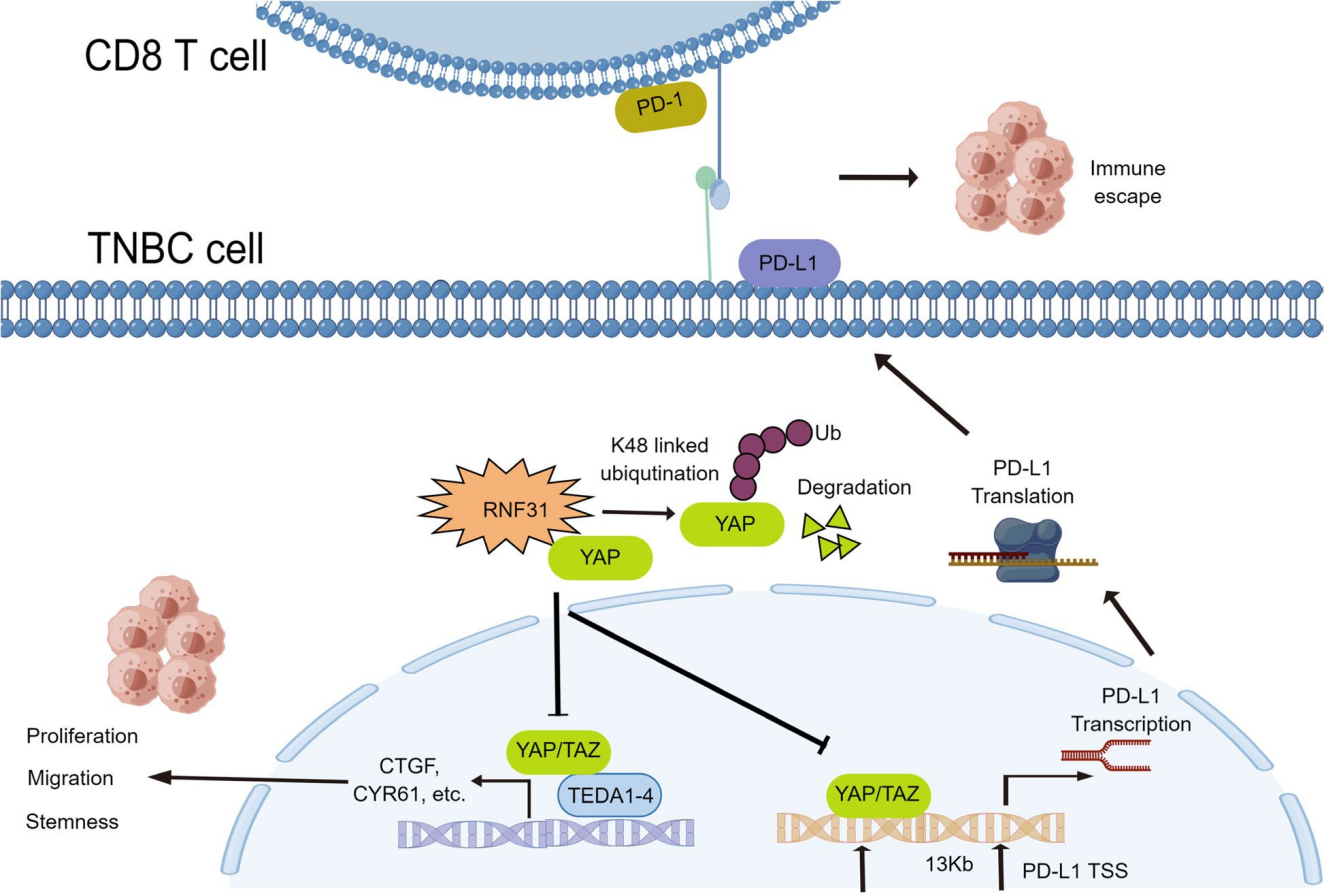
Schematic illustration of RNF31 represses cell progression and immune evasion via YAP/PD-L1 suppression in Triple Negative Breast Cancer (by Figdraw). RNF31 could associate with YAP protein and promote YAP protein K48-linked poly-ubiquitination and degradation, which subsequently inhibited YAP-driven signaling function and TNBC progression. Besides, RNF31 could also prevent tumor immune evasion via inhibiting Hippo/YAP/PD-L1 axis

In the current study, we find RNF31, which was reported to facilitate ER positive breast cancer cell growth via activating estrogen signaling and suppressing P53 signaling in our previously studies [11, 12], functions as a tumor suppressor in TNBC. RNF31 is decreased in TNBC samples, correlated with longer survival, and reversely correlated with YAP protein level in TNBC tumors. The functional analysis reveals RNF31 inhibited TNBC cell growth, migration, and metastasis in vivo and in vitro. The biological study showed that RNF31 could associate with YAP, promote YAP K48-linked poly-ubiquitination and degradation, which subsequently inhibited Hippo target gene activation. Interestingly, RNF31 is also identified as a novel modulator in TNBC immuno-response via suppression Hippo/YAP/PD-L1 axis (Fig. 7). Our study implicated a novel link with LUBAC complex with Hippo signaling in TNBC and a multi-faced function of RNF31 in cancer biology.

Hippo signaling pathway, which was originally found in fruit fly in controlling organ size, is highly conserved in several species [28, 29]. Hippo pathway maintains tissue hemostasis via regulation cell proliferation and differentiation. As an inhibitory pathway, MST1/2 phosphorylate LATS1/2, which subsequently phosphorylates and inactivates transcriptional co-activator YAP/TAZ. If the upstream phosphorylation cascade is not active, un-phosphorylated YAP/TAZ will interact with several transcriptional factors and facilitate several genes that promote cell proliferation and invasion [30]. In human cancers, the Hippo/YAP axis is always over-activated, such as TNBC [31, 32]. Recent studies have shown that high expression of YAP is associated with aggressive tumor behavior, as well as a biomarker and potential therapeutic target for poor prognosis in breast cancer patients, especially TNBC patients [33–35]. Besides, several ubiquitin ligases or deubiquitinases are also reported to modulate YAP stability and YAP activation in human cancers [21, 36]. Our previous studies showed that SHARPIN [37] and RNF187 [38] could promote YAP degradation and inhibit tumor progression. Here, we reported an important LUBAC component RNF31 in modulating breast cancer progression and Hippo signaling, which provide novel knowledge in crosstalk between LUBAC complex and Hippo pathway.

PD-L1 (Programmed death-ligand 1, CD274) is a trans-membrane protein, which suppresses the adaptive immune response. PD-L1 associates with PD1 and transmits the inhibitory signaling, which simultaneously reduces the proliferation of antigen-specific T cells and enhances the function of regulatory T cells. Several studies reported that PD-L1 was elevated in several human cancers, which facilitated tumor evasion [25, 39]. Besides, survival analysis showed that PD-L1 expression correlated with poor survival in several cancers, indicating its critical function in cancer progression [25]. Although immunotherapy has been proved effective in several human cancers, the specific biomarkers for PD-L1 blockage therapy are still not totally clear. The expression of PD-L1 is subject to several pathway regulations, including interferon pathway and NF-κB signaling [40]. The activation programmed cell death (PD)-1 pathway, which mediates cancer immune evasion, has become an attractive therapeutic target in TNBC [41]. Several studies have shown that the expression level of PD-L1 in TNBC cells correlates with immunotherapy response and overall prognosis [41–43]. Interestingly, our RNA sequencing data showed that RNF31 could be a possible inhibition factor for PD-L1 in TNBC. Molecular studies showed that RNF31 could suppress PD-L1 expression via inhibiting Hippo/YAP axis. Thus, modulating RNF31 function or enhance RNF31 expression could inhibit TNBC progression via dual mechanisms.

Our study reveals that RNF31 plays an inhibitory role of RNF31 in TNBC progression, which is dramatically different with ER positive breast cancer. RNF31 could associate with YAP protein and increase YAP protein K48-linked poly-ubiquitination and degradation, which inhibited YAP-driven signaling function and TNBC progression subsequently. Besides, RNF31 could also prevent tumor immune evasion via inhibiting Hippo/YAP/PD-L1 axis. Pharmaceutically activation RNF31 function or inducing RNF31 expression could be a promising strategy for TNBC treatments.

## Supplementary Information

**Additional file 1 Supplementary Fig. 1 A** Western blot detecting of RNF31 expression in HS578T cell exposed to indicated methods. **B-C** Transwell assay (left panel) of HS578T cells. Right panel shows quantification of transwell assay results. Scale bar 100 μm. **D-E** FACS analysis (left panel) was performed on the HS578T cell to detect the proportion of CD44 + CD24-cells. **F-G** FACS analysis (left panel) was performed on the HS578T cell to detect the proportion of apoptotic cells. The cells were incubated with PI and Annexin V. Right panel shows quantification of apoptosis proportion. **H-I** Wound healing assay (left panel) of HS578T cell migration capability following transfected with indicated treatment. **J-K** Representative images (left panel) of EdU assays in HS578T cell transfected with indicated treatment. EdU-positive cells, red; cell nuclei, blue. Right panel shows quantification of Edu results. Scale bar 100 μm.

**Additional file 2 Supplementary Fig. 2 A** FPKM expression form RNA-Seq in MDAMB231 cell (GSE218406). **B** Western blot detecting of p38、p-p38 and RNF31 expression in MDAMB231 cell exposed to indicated methods. **C** Western blot detecting of YAP、YAP P-S127 and RNF31 expression in HS578T cell exposed to indicated methods. **D** RT–qPCR results of CTGF and CYR61 mRNA expression in HS578T cell transfected with either indicated treatment. **E** Measurement of TEAD transcriptional activity using luciferase assays using reporters that contain tandem TEAD binding sites in HS578T cell. **F-H** RT–qPCR results of YAP mRNA expression in indicated cells transfected with either indicated treatment.

**Additional file 3 **Supplementary Fig. 3 **A** Western blot detecting of YAP expression in MDAMB231 cell exposed to indicated Verteporfin concentration for 12 h. **B-C** and **J-K** Transwell assay (left panel) of MDAMB231 cells. Right panel shows quantification of transwell assay results. Scale bar 100 μm. **D-E** and **L-M** FACS analysis (left panel) was performed on the MDAMB231 cell to detect the proportion of apoptotic cells. The cells were incubated with PI and Annexin V. Right panel shows quantification of apoptosis proportion. **F-G** and **H-I** Representative images (left panel) of EdU assays in MDAMB231 cell transfected with indicated treatment. EdU-positive cells, red; cell nuclei, blue. Right panel shows quantification of Edu results. Scale bar 100 μm.

**Additional file 4 Supplementary Fig. 4 A-B** Western blot detecting of YAP and RNF31 protein localized in cytoplasm and nucleus in the MDAMB231 cell with indicated treatment. Subcellular protein fractionation kit was used for cytoplasm and nucleus separation. Tubulin and Histone3 were engineered to cytoplasm and nucleus controls. Nucleus/Cytoplasm ratio of YAP protein (**B**). **C** Representative immunoblots to show the interaction between RNF31 with YAP or TAZ by immunoprecipitation (IP) with RNF31 antibody.

**Additional file 5 Supplementary Fig. 5 A** and **E** Western blot detecting of PD-L1 and RNF31 expression in BT549 and HS578T cells with indicated treatment. **B** and **F** RT–qPCR results of PD-L1 mRNA level with indicated treatment in BT549 and HS578T cells. **C-D** and **G-H** The cell-membrane localized PD-L1 in BT549 and HS578T cells with indicated treatment were analyzed by Flow cytometry using PD-L1 antibody.

## Data Availability

Publicly available data can be found in the GEO database (GSE218406). The original data are provided in supplementary materials.

## Abbreviations

TNBC: Triple negative breast cancer
RNF31: Ring finger protein 31
UBA: Ubiquitin binding associated domain
RBR: RING-between-RING domain
RING: Really interesting new gene
YAP: Yes-associated protein
HER2: Human Epidermal Growth Factor Receptor 2
TEAD: TEA domain transcriptional factor
LUBAC: Linear ubiquitin assembly complex
LATS: Large tumor suppressor kinase
PD-L1: Programmed death-ligand 1
TA domain: Transcription activation domain
