# Supplementary figures and images for: RNF31 represses cell progression and immune evasion via YAP/PD-L1 suppression in triple negative breast Cancer
Source: J Exp Clin Cancer Res. 2022 Dec 29;41:364. doi: 10.1186/s13046-022-02576-y (PMC9801641; doi:10.1186/s13046-022-02576-y)

Supplementary Figure 1

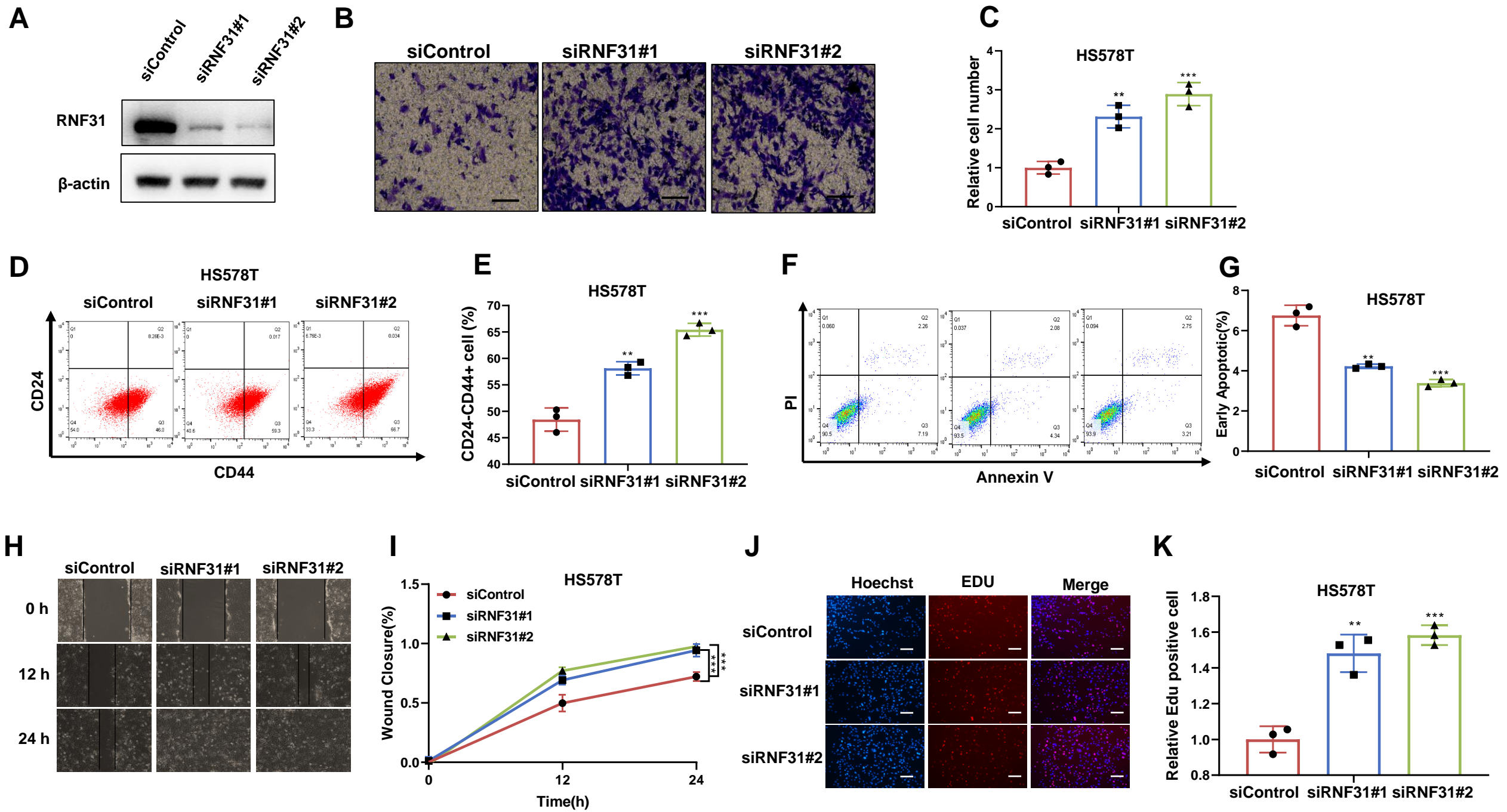

Supplement: Supplementary file 1 — Additional file 1 Supplementary Fig. 1 A Western blot detecting of RNF31 expression in HS578T cell exposed to indicated methods. B-C Transwell assay (left panel) of HS578T cells. Right panel shows quantification of transwell assay results. Scale bar 100 μm. D-E FACS analysis (left panel) was performed on the HS578T cell to detect the proportion of CD44 + CD24-cells. F-G FACS analysis (left panel) was performed on the HS578T cell to detect the proportion of apoptotic cells. The cells were incubated with PI and Annexin V. Right panel shows quantification of apoptosis proportion. H-I Wound healing assay (left panel) of HS578T cell migration capability following transfected with indicated treatment. J-K Representative images (left panel) of EdU assays in HS578T cell transfected with indicated treatment. EdU-positive cells, red; cell nuclei, blue. Right panel shows quantification of Edu results. Scale bar 100 μm. [file 13046_2022_2576_MOESM1_ESM.pdf]

Supplementary Figure 2

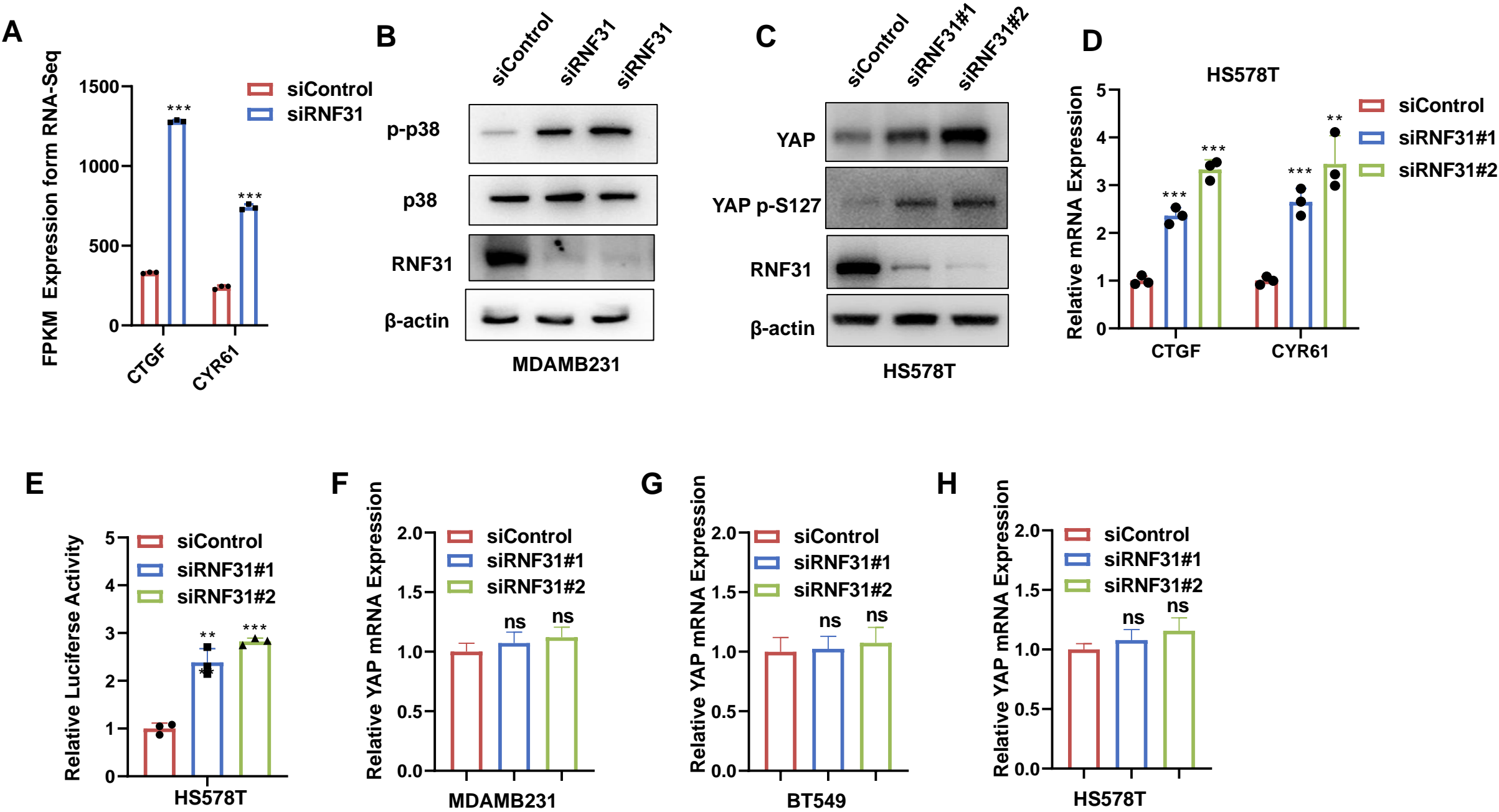

Supplement: Supplementary file 2 — Additional file 2 Supplementary Fig. 2 A FPKM expression form RNA-Seq in MDAMB231 cell (GSE218406). B Western blot detecting of p38、p-p38 and RNF31 expression in MDAMB231 cell exposed to indicated methods. C Western blot detecting of YAP、YAP P-S127 and RNF31 expression in HS578T cell exposed to indicated methods. D RT–qPCR results of CTGF and CYR61 mRNA expression in HS578T cell transfected with either indicated treatment. E Measurement of TEAD transcriptional activity using luciferase assays using reporters that contain tandem TEAD binding sites in HS578T cell. F-H RT–qPCR results of YAP mRNA expression in indicated cells transfected with either indicated treatment. [file 13046_2022_2576_MOESM2_ESM.pdf]

Supplementary Figure 3

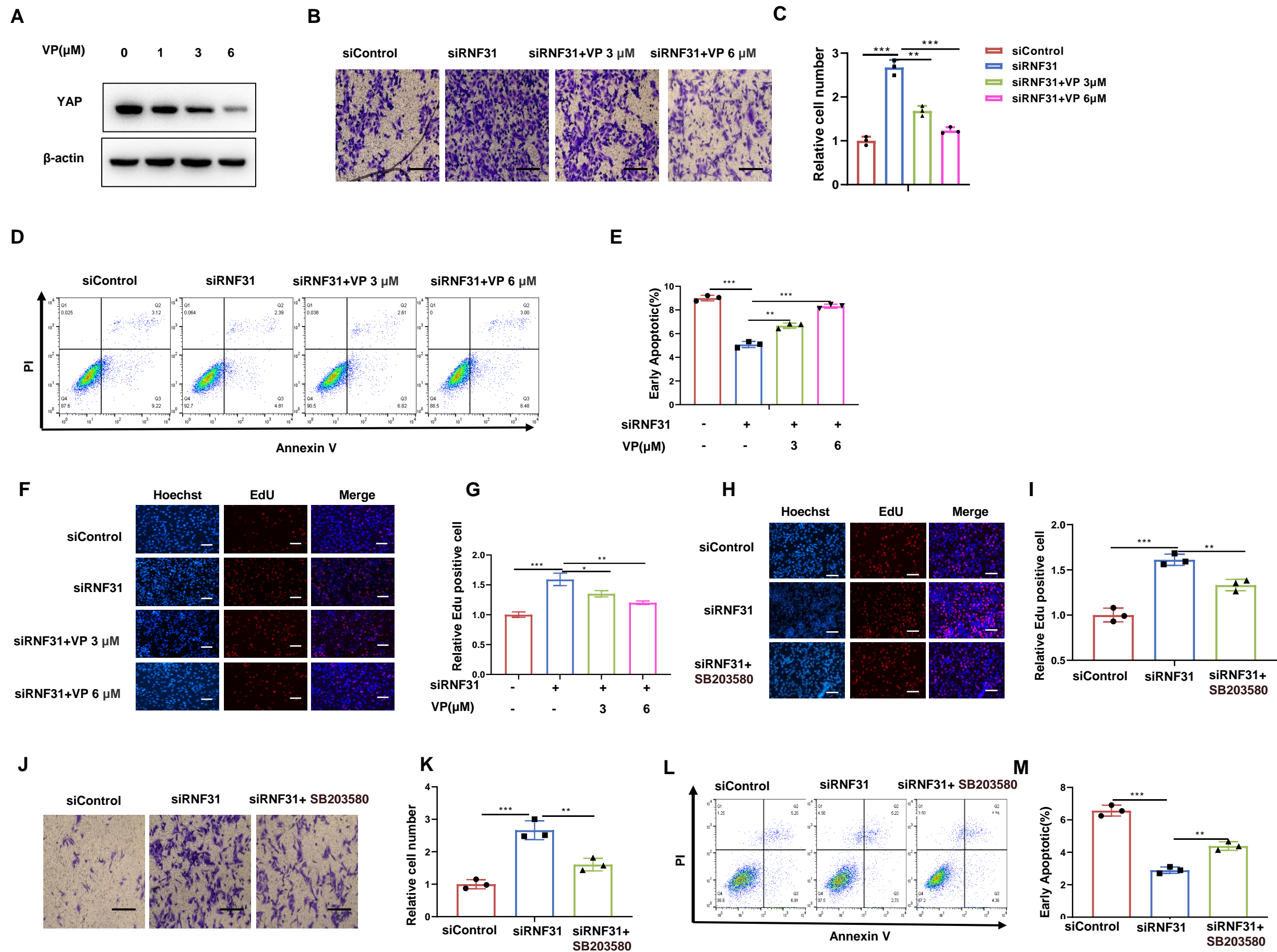

Supplement: Supplementary file 3 — Additional file 3 Supplementary Fig. 3 A Western blot detecting of YAP expression in MDAMB231 cell exposed to indicated Verteporfin concentration for 12 h. B-C and J-K Transwell assay (left panel) of MDAMB231 cells. Right panel shows quantification of transwell assay results. Scale bar 100 μm. D-E and L-M FACS analysis (left panel) was performed on the MDAMB231 cell to detect the proportion of apoptotic cells. The cells were incubated with PI and Annexin V. Right panel shows quantification of apoptosis proportion. F-G and H-I Representative images (left panel) of EdU assays in MDAMB231 cell transfected with indicated treatment. EdU-positive cells, red; cell nuclei, blue. Right panel shows quantification of Edu results. Scale bar 100 μm. [file 13046_2022_2576_MOESM3_ESM.pdf]

Supplementary Figure 4

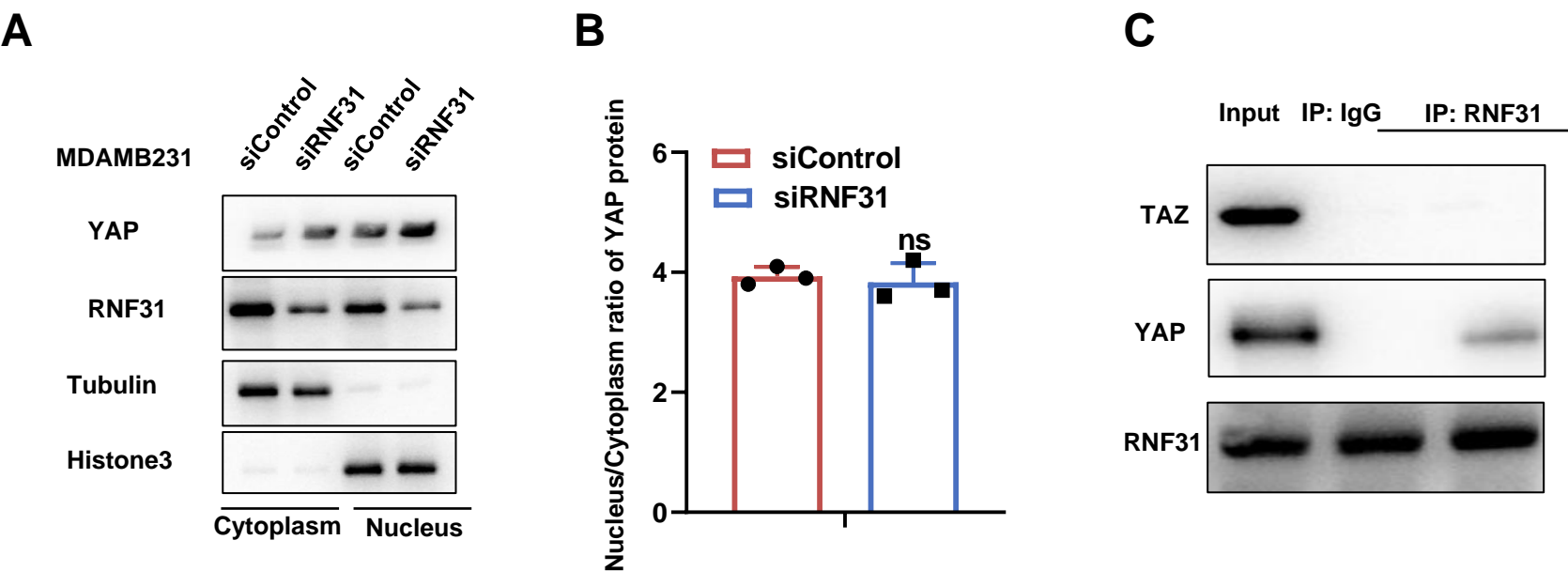

Supplement: Supplementary file 4 — Additional file 4 Supplementary Fig. 4 A-B Western blot detecting of YAP and RNF31 protein localized in cytoplasm and nucleus in the MDAMB231 cell with indicated treatment. Subcellular protein fractionation kit was used for cytoplasm and nucleus separation. Tubulin and Histone3 were engineered to cytoplasm and nucleus controls. Nucleus/Cytoplasm ratio of YAP protein (B). C Representative immunoblots to show the interaction between RNF31 with YAP or TAZ by immunoprecipitation (IP) with RNF31 antibody. [file 13046_2022_2576_MOESM4_ESM.pdf]

Supplementary Figure 5

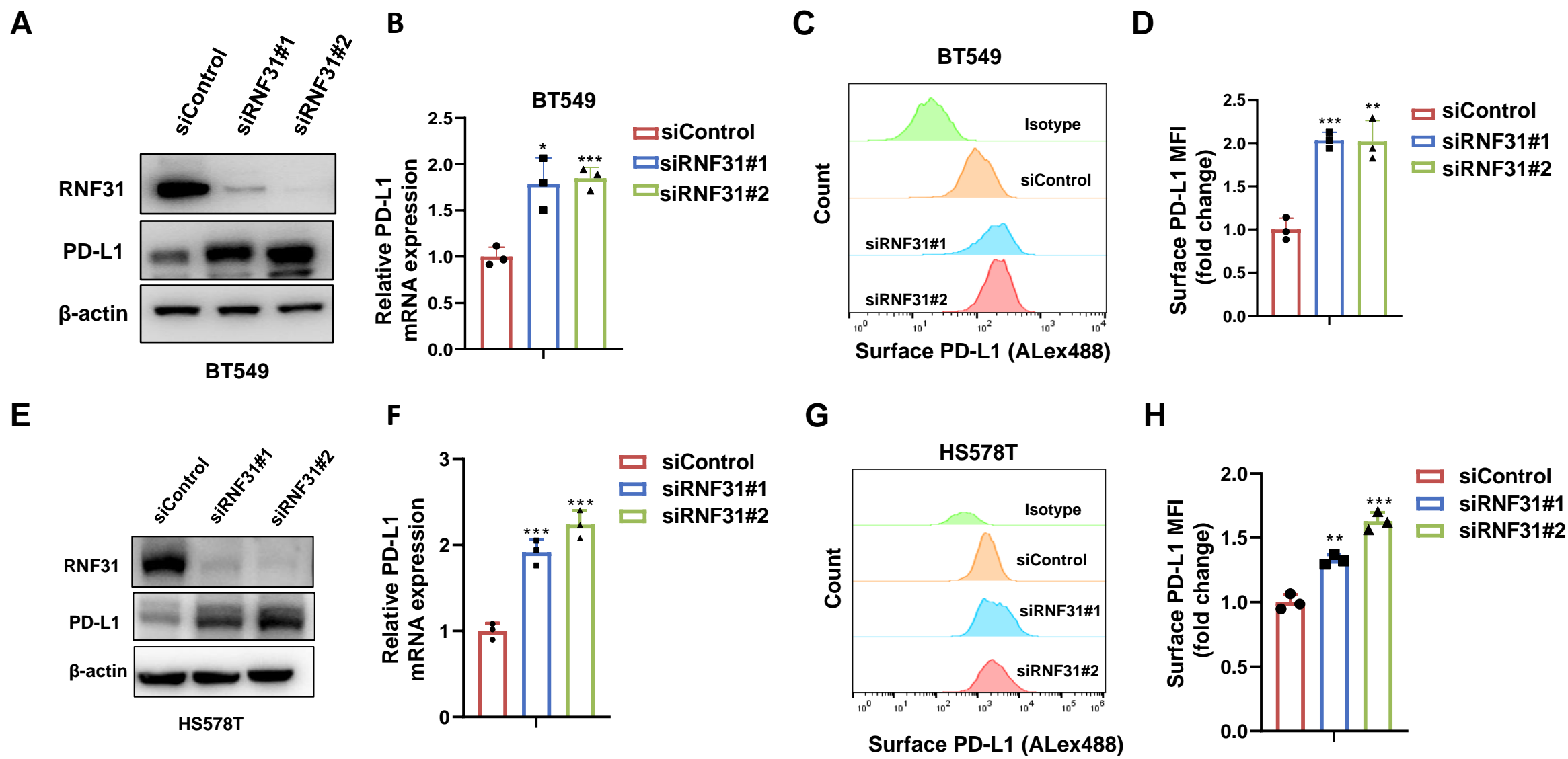

Supplement: Supplementary file 5 — Additional file 5 Supplementary Fig. 5 A and E Western blot detecting of PD-L1 and RNF31 expression in BT549 and HS578T cells with indicated treatment. B and F RT–qPCR results of PD-L1 mRNA level with indicated treatment in BT549 and HS578T cells. C-D and G-H The cell-membrane localized PD-L1 in BT549 and HS578T cells with indicated treatment were analyzed by Flow cytometry using PD-L1 antibody. [file 13046_2022_2576_MOESM5_ESM.pdf]
